# Mutational analysis in sodium-borate cotransporter SLC4A11 in consanguineous families from Punjab, Pakistan

**DOI:** 10.1371/journal.pone.0273685

**Published:** 2022-08-29

**Authors:** Afia Iqbal, Shagufta Naz, Haiba Kaul, Saima Sharif, Aysha Khushbakht, Muhammad Asif Naeem, Mehwish Iqtedar, Afshan Kaleem, Sabika Firasat, Farkhanda Manzoor

**Affiliations:** 1 Department of Zoology, Lahore College for Women University, Lahore, Pakista; 2 Department of Animal Breeding and Genetics, Genetics Discipline, University of Veterinary and Animal Sciences, Lahore, Pakistan; 3 Vision Impairment Lab of Genetic Diseases Group, Center of Excellence in Molecular Biology, Lahore, Pakistan; 4 Department of Biotechnology, Lahore College for Women University, Lahore, Pakistan; 5 Department of Biological Sciences, Quaid-e-Azam University, Islamabad, Pakistan; Government College University Faisalabad, PAKISTAN

## Abstract

**Aim:**

To identify the molecular basis of Congenital Hereditary Endothelial Dystrophy CHED caused by mutations in SLC4A11, in the consanguineous Pakistani families.

**Methods:**

A total of 7 consanguineous families affected with Congenital Hereditary Endothelial Dystrophy were diagnosed and registered with the help of ophthalmologists. Blood samples were collected from affected and unaffected members of the enrolled families. Mutational analysis was carried out by DNA sequencing using both Sanger and Whole Exome Sequencing (WES). Probands of each pedigree from the 7 families were used for WES. Results were analyzed with the help of different bioinformatics tools.

**Results:**

The sequencing results demonstrated three known homozygous mutations in gene SLC4A11 in probands of 7 families. These mutations p.Glu675Ala, p.Val824Met, and p.Arg158fs include 2 missense and 1 frameshift mutation. The mutations result in amino acids that were highly conserved in SLC4A11 across different species. The mutations were segregated with the disease phenotype in the families.

**Conclusion:**

This study reports 3 mutations in 7 families. One of the pathogenic mutations (p.R158fs) was identified for the first time in the Pakistani population. However, two mutations (p.Glu675Ala, p.Val824Met) were previously reported in two and one Pakistani family respectively. As these mutations segregate with the disease phenotype and bioinformatics tool also liable them as pathogenic, they are deemed as probable cause of underlying disease.

## Introduction

According to a National blindness survey, 11.8% of blindness in Pakistan is caused by corneal opacity [1]. Approximately 80% of the world’s blind population lives in developing countries, of which 8%-25% have some form of corneal disease. Of the corneal diseases, congenital hereditary endothelial dystrophy CHED is more common in developing countries with some form of inbreeding [2]. The actual incidence, prevalence, and sex distribution of CHED are not known to date [3]. The majority of the cases have been found, in Pakistan, India, Saudi Arabia, Myanmar (Burma), and Ireland, in children of consanguineous parents. More than 100 mutations in the SLC4A11 gene have been recognized so far, the majority of which are missense mutations with a few frameshift deletions [4, 7]. Congenital hereditary endothelial dystrophy (Phenotype MIM number: 217700) is characterized by bilateral diffuse clouding of both corneas from infancy and it is a rare disease. Edward Maumenee, MD was the first scientist who described CHED in 1960. He reported a series of cases of varying corneal clouding that were congenital and mainly stationary [5]. In CHED, the corneal clouding ranges from a mild haze to ground glass, milky appearance and it can lead to amblyopia.

Using mapping techniques, researchers were able to correlate autosomal recessive inheritance of CHED to genetic markers within the 20p13 chromosomal locus [6]. This provided the first hint to the etiology of CHED. This assignment ultimately led to the identification of SLC4A11 gene mutations as the genetic cause of CHED. SLC4A11 encodes a membrane protein formerly known as "BTR1" (Bicarbonate Transporter Related protein 1), which indicates its membership in the SLC4 family of bicarbonate-transporting membrane proteins. Despite its name, the unique amino acid sequence of SLC4A11 distinguishes it from its family. Perhaps, as a result, robust transport is not one of the various molecular functions identified in SLC4A11 [7, 8]. SLC4A11 mutations are expected to harm neural crest cell proliferation and final differentiation into the endothelium monolayer [9]. The pathophysiology of CHED is linked to mutations in the SLC4A11 gene [10, 11]. At the time of writing, mutations in SLC4A11 have been identified in over a hundred CHED patients. Concerning the molecular action and function of SLC4A11 in healthy and sick corneas, however, there is still much to learn [12].

During this study families affected with CHED were identified through different eye hospitals in Punjab, Pakistan. Clinical ascertainment was done by a trained ophthalmologist. The diagnosis of the disease was confirmed by genetic analysis to find gene mutations. These mutations (p. Glu675Ala, p.Val824Met) [13–15] alter a highly conserved amino acid in the encoded SLC4A11 protein. To eliminate these variants as polymorphism, the ESP and gnomAD databases were checked along with 1000 Genomes Phase 3, from any population. Variants in the SLC4A11 gene were found in the East Asian population and showed the highest population MAF: <0.01, indicating that these are rare variants. The frameshift deletion [c.473-480del: p.Arg158fs] found may be caused by the truncation of protein and the addition of novel amino acids [16]. Most characterized SLC4A11 mutant proteins display the intracellular retention phenotype characteristic of misfolded membrane proteins [17]. The objective of this research was to identify the molecular basis of CHED in consanguineous families enrolled from Punjab, Pakistan. We report mutations in the SLC4A11 gene (NM_032034.3) segregating in consanguineous families. There are 3 known homozygous mutations in gene SLC4A11 in probands of 7 families. These mutations p.Glu675Ala, p.Val824Met include 2 missense mutations in 6 families and 1 frameshift deletion p.Arg158fs in 1 family [13–15].

Paternal consanguinity is linked to more than half of the cases recorded and is consistent with autosomal recessive inheritance [14]. Consanguineous parents and a family history of CHED enhance the likelihood of a neonate contracting the disease. Due to a 25% chance of recurrence in future generations, it is suggested that families with children diagnosed with CHED receive genetic counseling [3].

## Subjects and methods

### Clinical ascertainment

A total of 7 consanguineous families with affected members having CHED was registered for molecular analysis according to the ethical committee of the Institutional Review Board of the Lahore College for Women University, Lahore (LCWU). Pakistan. Written Consent was obtained after informing the family and for the minors, consent was obtained from their parents and guardians. The affected status was determined for each study participant using the slit-lamp examination. Elaborated family and medical histories were obtained to evaluate the familial pattern and to omit any other health conditions segregating with the disease. All the enrolled patients had CHED which was described as bilateral corneal clouding at the time of birth or in infancy. Patients suffered from symptoms that include reduced vision, often leading to amblyopia. On slit-lamp evaluation, thickening of Descemet’s membrane and diffuse corneal edema affecting both eyes were detected. It is the hallmark of CHED and was present in both eyes of patients mostly at birth. The visual aspect of blue-gray ground glass varied from corneal edema to total corneal opacification. Pedigrees of all the families were drawn and blood samples were taken for DNA isolation from the patients and their relatives.

### DNA extraction from blood

Genomic DNA was isolated from blood samples according to a standardized protocol used in Lahore College for Women University, Lahore (LCWU) [18], which includes: Frozen blood samples were thawed in warm water for RBC lysis. T.E buffer (10 mM Tris HCl + 0.1 mM EDTA) was added and shaken vigorously. Centrifuged at 3000 rpm at 25°C for 25 to 30 minutes. Three to four washings were given to completely remove hemoglobin and attain a pellet of WBC. According to the volume of blood samples, TNE buffer (10 mM Tris HCl + 0.1 mM EDTA + 400 mM NaCl) in (6 ml/10 ml of blood), 10% SDS and Proteinase K (50 μl 10 mg/ml) were added for the digestion of proteins in pellets of WBCs. Samples were then incubated at 37°C in a shaker overnight. Chilled 6 M NaCl (1 ml/10 ml of blood) was added and shaken till it becomes foamy. Again placed at low temperature for another 10–15 minutes and Phenol Chloroform Isoamylalcohol (PCI) (25:24:1 mixture) in (2ml/ 10ml of blood) was added. Centrifuged at 3000 rpm and 4°C for 25 minutes to pellet down the salts and proteins. Then isopropanol was added with equal volume to the aqueous solution, centrifuged at 3000 rpm, and at 25°C for 20 minutes. Washed the DNA pellet with 70% ethanol (5ml/10ml of blood). Added low TE (10 mM Tris HCl + 0.1 mM EDTA) buffer in dried pellet for DNA storage (1.5 ml/10ml of blood) [18].

### Mutational analysis

All 20 exons of SLC4A11 (NM_032034) were screened using Sanger sequencing. Initially, 4 probands from 4 families were sequenced. The primer pairs were designed with the help of using Primer3 Plus software. Genomic DNA of 50ng was amplified in a reaction volume of 25 μl containing ready-to-use solution PCR master mix (Thermo) 2X with 5 units/ul Taq DNA polymerase, 400 μM each dNTPs, 3 mM MgCl_2_ and reaction buffers at optimal concentrations. The polymerase chain reaction was performed with an initial denaturation at 94°C for 5 minutes followed by denaturation at 94°C for 30 seconds, annealing for 30 seconds at 65°C with the decrease of 1°C after each cycle (10 cycles) and then extension at 72°C for 30 seconds; each followed by 30 cycles of denaturation at 94°C for 30 seconds, annealing at 55°C for 30 seconds and extension at 72°C for 30 seconds. The final extension was performed at 72 ˚C for 10 minutes and the final hold at 4 ˚C. PCR amplicons were analyzed on 1.5% agarose gel and purified by ethanol precipitation. Purified products were subsequently sequenced using the BigDye terminator version 3.1 Cycle Sequencing Kit (Applied Biosystems) with one of the original PCR primers according to the manufacturer’s instructions. SeqScape v2.5 and Sequencing Analysis version 5.2 (Life Technologies Ltd) software were used to analyze the resulting sequence data.

Whole Exome Sequencing (WES) was performed in probands from seven families by using the Twist Comprehensive Exome Panel (Twist bioscience) and sequenced on a HiSeq 4000 instrument (Illumina) with an average coverage of 100–120X at each nucleotide position. Raw reads were mapped to the human genome reference sequence using Novoalign software (V3.08.00; Novocraft Technologies, Selangor, Malaysia) (build hg19). Base Quality score recalibration was done by HaplotypeCaller (GATK, v.4.0.3.0). Duplicates were removed using Picard (v. 2.14.0-SNAPSHOT). GATK (GATK v4.0) was used to find small indels and SNVs [19]. Automap [20] was used to achieve homozygosity mapping.

Different validating tools were used to evaluate the damaging/pathogenic effects of the mutations on the structure and function of a protein. Conservation of particular amino acids in different species is one of the most important parameters to check, so it was also analyzed through the UCSC genome browser. The frequency of all the variants was analyzed through the gnomAD database (https://gnomad.broadinstitute.org/) to further validate the conservation of the affected amino acid. Most likely damaging mutations were then confirmed with different bioinformatics programs: Polyphen2 (Polymorphism Phenotyping) [21] (http://genetics.bwh.harvard.edu/pph2), SIFT (Sorting Intolerant from Tolerant) (http://sift.jcvi.org) and PROVEAN (http://provean.jcvi.org/index.php) (Protein Variation Effect Analyzer) for determining variant pathogenicity and predict whether a variant has an impact on the biological function of the protein [22].

## Results

Seven consanguineous families (PKCD-AI01, PKCD-AI02, PKCD-AI03, PKCD-AI04, PKCD-AI06, PKCD-AI07, and PKCD-AI09) diagnosed with CHED were enlisted for this investigation. All the families were residents of Punjab, Pakistan.

**Fig 1:**
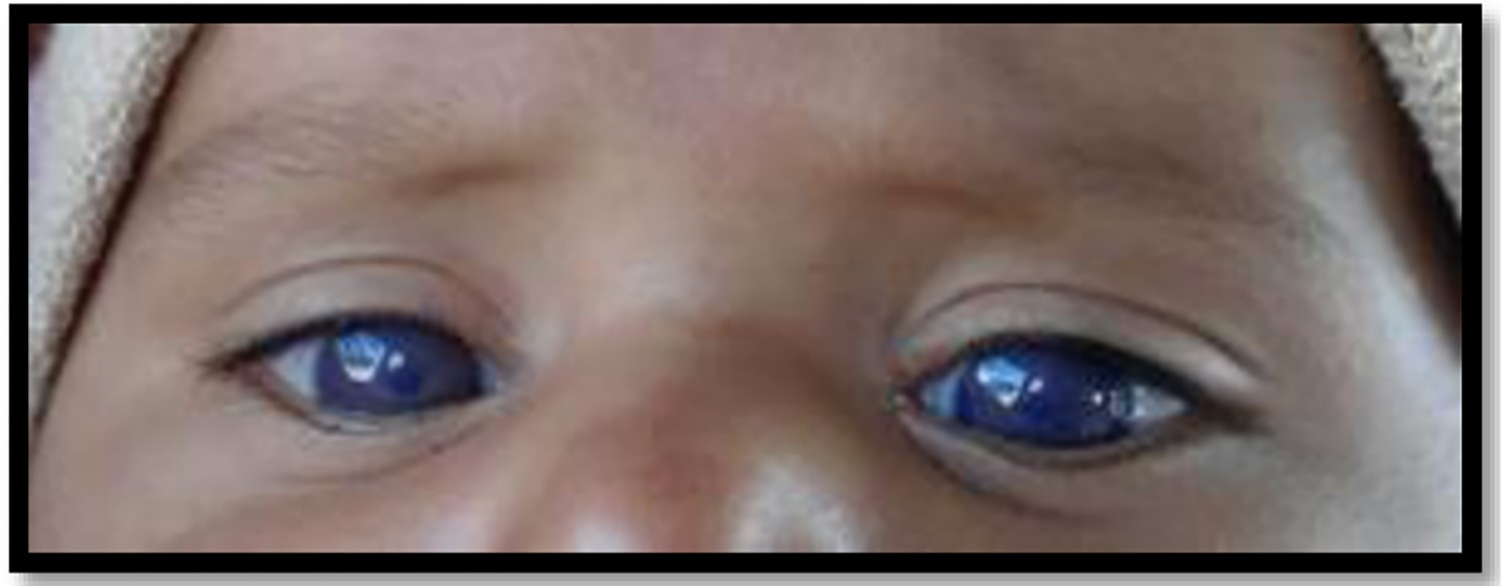
Affected individual of family PKCD-AI03 with CHED depicting bilateral hazy corneas.

Overall, a total of 56 individuals comprising 24 affected and 32 normal members were included in this study. Details about the pedigree structure and information are tabulated in Table 1. The clinical and ophthalmic assessment was done for the patients and there was no other allied anomaly segregating with CHED phenotypic. Clinical assessment and medical reports revealed that all the patients of the enrolled families had swollen and bilateral opaque corneas at the time or soon after birth (Fig 1). Pedigrees were drawn up to the third generation which showed that all the families were highly inbred with consanguineous marriages. The mode of inheritance was found to be autosomal recessive with all the parents of diseased individuals not having the CHED phenotype.

**Table 1:** Mutations found in *SLC4A11* gene from residents of Pakistani families.

Family ID	Ethnicity/caste	Mutation	Protein change	Nature of mutation and Zygosity
PKCD-AI01	Punjabi/ Arain	c.2024A>C	p.Glu675Ala	Missense /Homozygous
PKCD-AI02	Punjabi/ Ansari			
PKCD-AI04	Punjabi/ Arain			
PKCD-AI06	Punjabi/ Jutt			
PKCD-AI07	Punjabi/ Kamboh			
PKCD-AI03	Punjabi/ Arain	c.2470G>A	p.Val824Met	Missense /Homozygous
PKCD-AI09	Saraiki/ Awan	c.473_480del	p.Arg158fs	Frameshift /Homozygous

**Fig 2:**
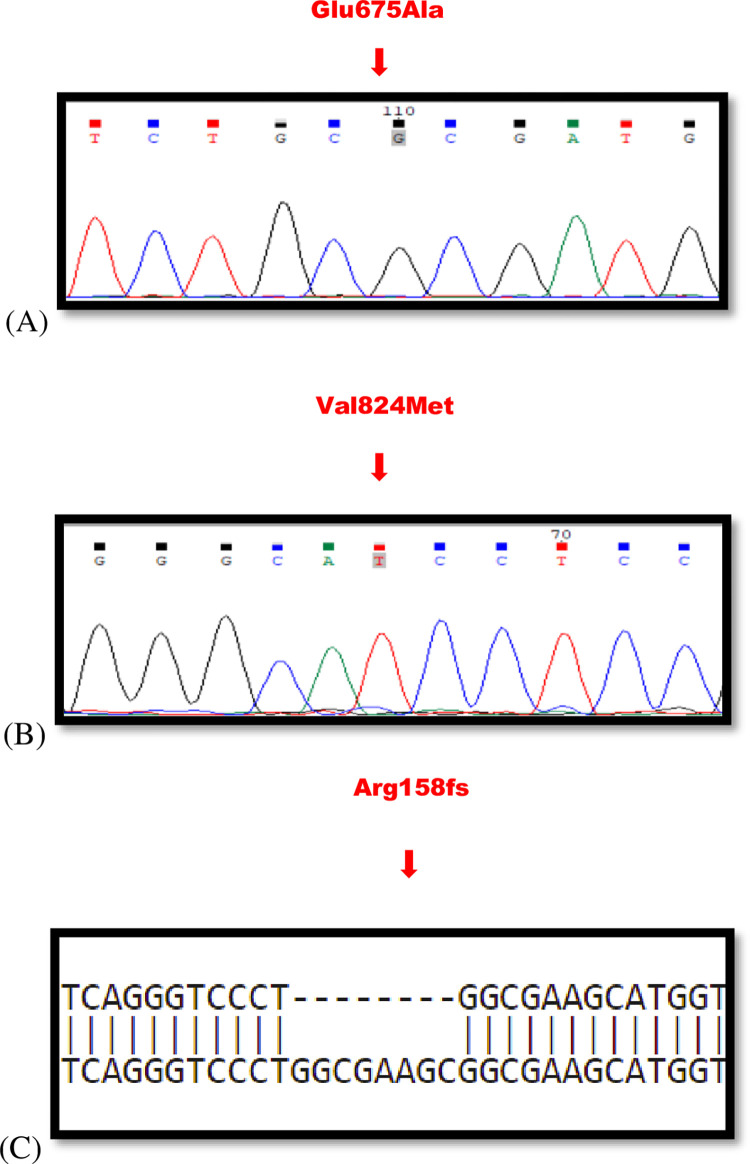
Mutations in the nucleotide sequence of SLC4A11 gene producing CHED phenotype. **(A)** Substitution of T>G in the nucleotide sequence of SLC4A11 gene producing CHED phenotype. The chromatograms of the patients are shown from families PKCD-AI01, PKCD-AI02, PKCD-AI04, PKCD-AI06, and PKCD-AI07. An arrow signifies homozygous T>G change causing missense mutation resulting in amino acid replacement of glutamic acid at location 675 with alanine. **(B)** Substitution of C>T in the nucleotide sequence of SLC4A11 gene producing CHED phenotype. The chromatograms of the patients are shown from families PKCD-AI03. An arrow signifies homozygous C>T change causing missense mutation resulting in amino acid replacement of valine at location 824 with methionine. **(C)** Frame shift deletion in SLC4A11 gene producing CHED phenotype in family PKCD-AI09 at location Arginine 158 causing truncation of protein and addition of novel amino acids.

**Fig 3:**
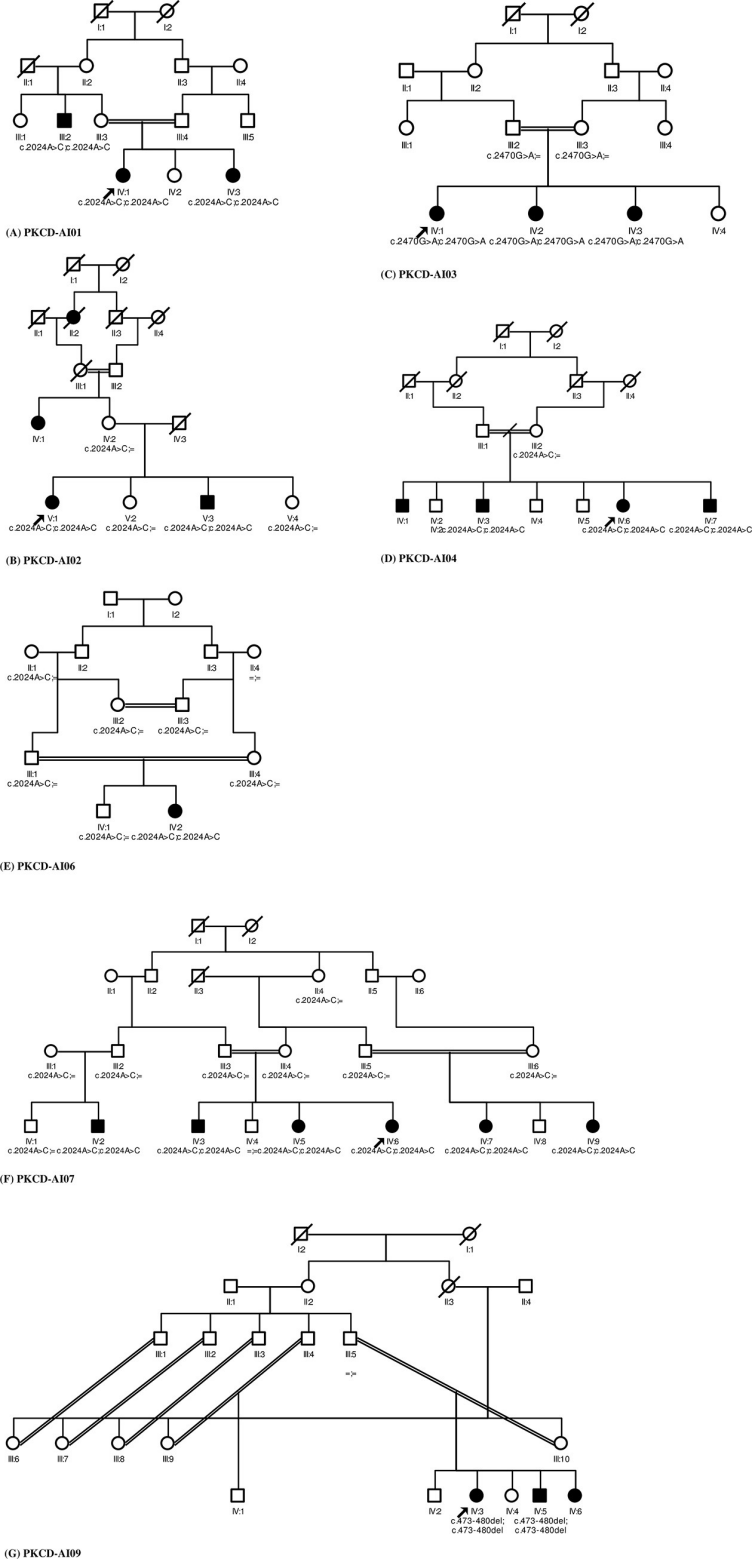
Pedigree chart of families diagnosed with CHED. Dark circled symbols specify affected persons, Bright circled symbols specify unaffected persons or persons with undefined disease status. **(A**) Pedigree of PKCD-AI01 family, affected with ar CHED. (**B**) Pedigree of PKCD-AI02 family, affected with ar CHED. (**C**) Pedigree chart of PKCD-AI03 family, affected with ar CHED. (**D**) Pedigree chart of PKCD-AI04 family, affected with ar CHED. (**E**) Pedigree chart of PKCD-AI06 family, (**F**) Pedigree chart of PKCD-AI07 family. (**G**) Pedigree chart of PKCD-AI09 family. Black arrows on the left bottom display the probands from each family. Individuals with homozygous mutations are symbolized as [mutation;mutation], heterozygous carriers as [mutation; =] and non-carriers individuals homozygous for the healthy allele as =, =.

The results of DNA sequencing (WES) on the probands of all 7 families showed 2 missense mutations (1 missense mutation shared by five probands) and 1 frameshift deletion (one proband) in a total of 7 families (Table 1). All three mutations were previously identified in different populations. Two of the missense mutations leading to p.Glu675Ala and p.Val824Met were previously reported in the Pakistani population [13, 15] while the remaining one frameshift mutation [14] was not detected in Pakistani patients before this study. The missense mutation (c.2024A>C: p.Glu675Ala) in the SLC4A11 gene, was identified in 5 unrelated families. In all affected individuals homozygous c.2024A>C: p.Glu675Ala mutations (Fig 2) were identified in pedigrees PKCD-AI01, PKCD-AI02, PKCD-AI04, and PKCD-AI06, PKCD-AI07 (Fig 3). In healthy subjects from the family, the p.Glu675Ala mutation was not detected. A highly conserved amino acid (glutamic acid) was present in the SLC4A11 protein at position 675. The mutation replaced the amino with alanine with the GERP score of 5.08, which indicates the high pathogenicity of the variant.

Second homozygous (c.2470G>A: p.Val824Met) alteration (Fig 2) was found in the probands of family PKCD-AI03 (Fig 3) affecting the SLC4A11 gene. This missense change was also highly conserved among different species, signifying that mutation at this location would be deleterious as this variant have the GERP score of 5.37. The third mutation identified in this study was frameshift deletion c.473-480del leading to p.Arg158fsX4 change (Fig 2), found in affected individuals of pedigree PKCD-AI09 (Fig 3), and may be caused due to the truncation of protein and addition of novel amino acids. All three mutations were segregated with the disease phenotype in all the families examined.

## Discussion and conclusion

This study reports the three homozygous mutations in 7 highly inbred Pakistani families from Punjab province. Each proband from the affected family was subjected to WES and reported homozygous mutations were detected in 7 probands in SLC4A11. These involved two missense and one frameshift deletion in probands of 7 families. These include 2 missense mutations (p.Glu675Ala, p.Val824Met) in 6 families (PKCD-AI01, PKCD-AI02, PKCD-AI03, PKCD-AI04, PKCD-AI06, and PKCD0AI07) and 1 frameshift mutation (p.Arg158fs) in 1 family (PKCD-AI09).

**Fig 4:**
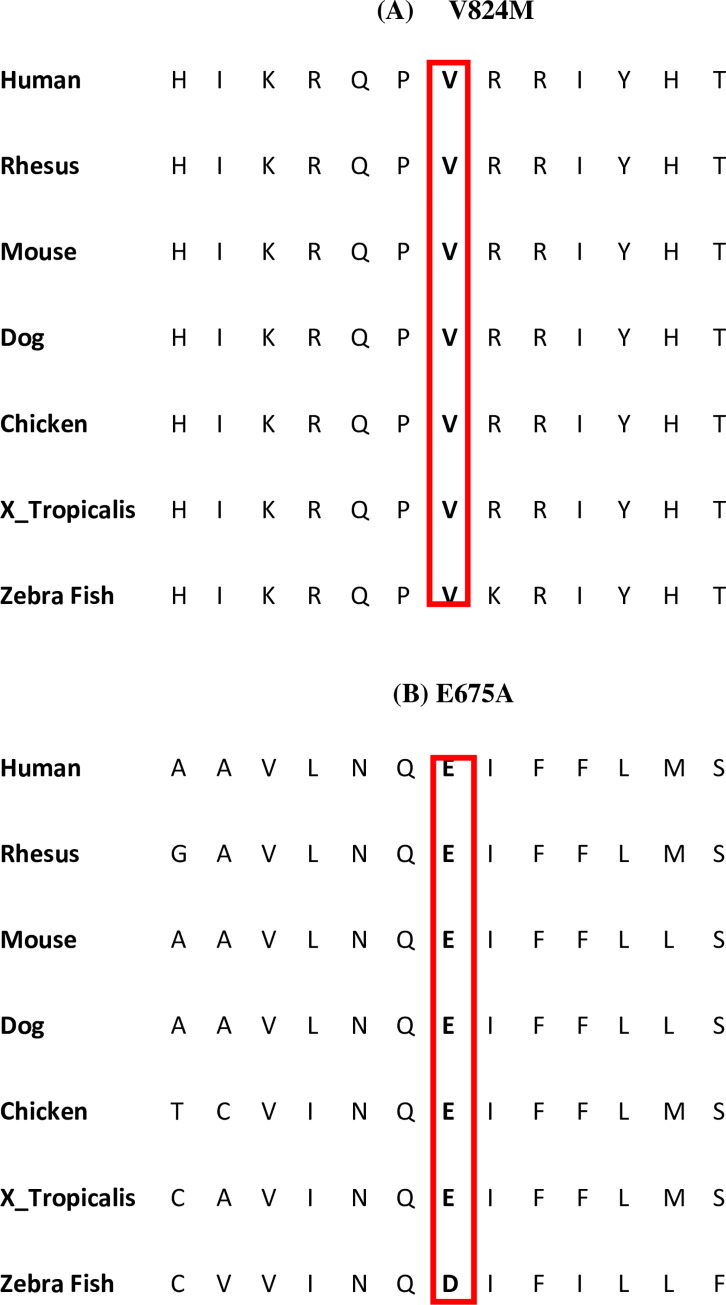
Protein sequence around missense mutations. (A) and (B) Alignments of protein sequence around missense mutations across different species representing high conservation of substituted amino acid. These substitutional changes lie in exon 18 and 15 (p.V824M, p.E675A) respectively.

The missense change p.Glu675Ala with c.2024A>C residue lies in the domain, transmembrane region with a range of 652 to 874 according to the SMART sequence analysis tool. As shown in Fig 4, this residue is highly conserved in a wide range of species which denotes that substitution of this amino acid would be pathogenic, and may produce a change in protein which leads to the occurrence of this specific corneal disease that is CHED. This identified mutation was previously reported in two families from Lahore, Pakistan [13]. However, in our study, the same trend was observed in five unrelated families living in cities across Punjab other than Lahore. Even though all of the families belonged to the same Punjabi ethnic group, they lived in different cities across Punjab and belonged to distinct castes. PKCD-AI01, PKCD-AI03, and PKCD-AI04 belonged to caste Arain but were residents of two separate cities i.e., Multan, Pattoki, and Sahiwal. PKCD-AI02, PKCD-AI06, and PKCD-AI07 belonged to the cities of Hafizabad, Burewala, and Farooqabad, respectively, from the Ansari, Jutt, and Kamboh castes. In some populations, rare genetic diseases have been recorded often. The founder effect is one of the mechanisms that could be hypothesized to explain the mutation found in these families. The substitution of glutamic acid at position 675 with alanine in SLC4A11 protein causes pathogenic effects on protein structure and function. This might be because glutamic acid is a negatively charged residue and is hydrophilic. However, it is substituted with a neutral, hydrophobic amino acid alanine [23].

The second homozygous missense mutation p.Val824Met found previously [15], was reported from Indian origin in 6 families while from Punjab, Pakistan the family PKCD-AI03 belonged to caste Arain. This substitutional change c.2470G>A resulting in (p.Val824Met) is present in domain 13 of the transmembrane region, extracellular Loop 7. Conservation, when checked, revealed that this residue is maintained in most of the species and substitution changes the sequence of the SLC4A11, producing a variant with disease-causing properties. According to our information, this mutation was not previously identified in Pakistani families but it has now been identified in one recent study [15]. Hence, both of these missense mutations alter a highly conserved amino acid in the encoded SLC4A11 protein.

Family PKCD-AI09 belonged to Saraiki ethnic group and was identified with a frameshift deletion comprising 8 base pairs (c.473_480delTGGCGAAGC, R158QfsX4), found first time in one family out 7 families from Punjab, Pakistan under observation. This deletion had previously been detected in two Indian and one Gipsy (Eastern European) ancestries [16]. Pathogenicity measure identified that this deletion is damaging and produces a change in the SLC4A11 gene, found to be the particular cause of congenital hereditary endothelial dystrophy.

Up till now, more than 100 mutations have been reported for the SLC4A11 gene and most of them are missense mutations and there are few frameshift deletions [7, 24]. Transmembrane protein is encoded by SLC4A11 which works as a bicarbonate transporter. Bicarbonate transporter-related protein-1 (BTR1) is an important protein as it transports Na and OH in the absence of borate. Its function is to pump fluid from the stroma of the eye to the aqueous humor actively by countering the osmotic gradient and keeping the cornea clear [25]. Alterations of the SLC4A11 gene in CHED may result in the loss of protein function and changed BTR1, incapable to approach the cellular membrane and work aptly [26]. As a result, there is disruption of collagen fibrils, stromal edema, and thickening of the Descemet’s membrane. The reason for this thickness is an abnormal secretion of the endothelium terminating in opacification of the cornea [27]. Although all the mutations reported in this study were identified previously in different families. One of the frameshift mutations is being reported for the first time in Pakistani kindred. It can be concluded that especially the missense mutation (p.E675A and p.V824M) can be due to the founder’s effect as they are identified in patients of other ancestry and ethnicity.

The present analysis recognized 3 changes from Punjab, Pakistan, adding to the repository of alterations in the SLC4A11 gene, and logged a high notch of genetic assortment in CHED. This information will be useful for providing rapid prenatal diagnosis and genetic counseling to families and their relatives.
