# Correction: Mutational analysis in sodium-borate cotransporter SLC4A11 in consanguineous families from Punjab, Pakistan

**DOI:** 10.1371/journal.pone.0293769

**Published:** 2023-10-26

**Authors:** Afia Iqbal, Shagufta Naz, Haiba Kaul, Saima Sharif, Aysha Khushbakht, Muhammad Asif Naeem, Mehwish Iqtedar, Afshan Kaleem, Sabika Firasat, Farkhanda Manzoor

There is an error in the affiliation for author Sabika Firasat. The correct affiliation is: Department of Zoology, Faculty of Biological Sciences, Quaid-i-Azam University, Islamabad.
